# Durable Response to the Combination of Atezolizumab With Platinum-Based Chemotherapy in an Untreated Non-Smoking Lung Adenocarcinoma Patient With *BRAF* V600E Mutation: A Case Report

**DOI:** 10.3389/fonc.2021.634920

**Published:** 2021-06-10

**Authors:** Xiaomin Niu, Yingjia Sun, David Planchard, Luting Chiu, Jian Bai, Xinghao Ai, Shun Lu

**Affiliations:** ^1^ Department of Shanghai Lung Cancer Center, Shanghai Chest Hospital, Shanghai Jiao Tong University, Shanghai, China; ^2^ Gustave Roussy, Department of Medical Oncology, Thoracic Unit, Villejuif, France; ^3^ Berry Oncology Corporation, Beijing, China

**Keywords:** non-small cell lung cancer, proto-oncogene protein B-raf, programmed death ligand-1, BRAF inhibitor, immunotherapy

## Abstract

**Background:**

Immune checkpoint inhibitor (ICPi) has become a major treatment in advanced non-small cell lung cancer (NSCLC) and demonstrated a clinical benefit for NSCLC patients with high programmed death ligand-1 (PD-L1) expression without *EGFR/ALK/ROS1* drivers; however, the benefit in *BRAF* V600E NSCLC is so far unknown. Here, we report a case of prolonged tumor response to the combination of immunotherapy with chemotherapy in a non-smoking *BRAF* V600E NSCLC patient.

**Materials and Methods:**

We verify a co-expression of *BRAF* V600E mutation and PD-L1 high expression more than 50% on formalin-fixed paraffin-embedded tumor sample of a newly diagnosed lung adenocarcinoma patient by immunohistochemistry and *BRAF* V600E/*EGFR*/*ALK*/*ROS1* Mutations Detection Kit. The tissue and liquid biopsies were further subjected to next-generation sequencing (NGS) for identification of mutations with progression on immunotherapy and BRAF inhibitor (BRAFi). The patient had provided written informed consent and authorized the publication of clinical case.

**Results:**

We demonstrate the case of 62-year-old female non-smoker with high PD-L1 expression and *BRAF* V600E mutated NSCLC. The progression-free survival (PFS) of first-line combination of atezolizumab with platinum-based chemotherapy and sequential second-line treatment with BRAFi Vemurafenib are 20 and 5.5 months, respectively.

**Conclusion:**

This case shows a durable response to ICPi in *BRAF* V600E non-smoking lung adenocarcinoma with PFS of 20 months under first-line atezolizumab plus chemotherapy treatment. The case supports the idea that the combination immunotherapy may be an attractive option for *BRAF* V600E mutated non-smoking NSCLC with high PD-L1 expression.

## Introduction

Approximately 2% of non-small cell lung cancer (NSCLC) harbors a *BRAF* mutation, and *BRAF* V600E mutation accounts for 50–70% of *BRAF* mutated lung adenocarcinomas. Recently, the European Medicines Agency (EMA) and the Food and Drug Administration (FDA) approved the combination of dabrafenib with trametinib as an effective treatment for *BRAF* V600E mutated patients. Another study reported an objective response rate (ORR) of 44.9% and a median progression-free survival (PFS) of 5.2 months in 100 *BRAF* V600 mutated NSCLC patients treated with Vemurafenib (1).

However, the question of the therapeutic options beyond BRAF inhibitor (BRAFi) remains a critical issue. Recently, FDA approved immune checkpoint inhibitor (ICPi) atezolizumab for *BRAF* V600 unresectable or metastatic melanoma based on the result of IMspire150 trial (NCT02908672) (2). NSCLC patients who had high programmed death ligand-1 (PD-L1) expression without *EGFR/ALK/ROS1* drivers and had benefited from ICPi have been reported, while, the benefit in *BRAF* V600E NSCLC is still unclear. IMMUNOTARGET registry study is to investigate the activity of ICPi across NSCLC harboring oncogenic alterations in 24 centers from 10 countries, the result showing that ORR was 24% and median PFS was 3.1 months for 43 *BRAF* NSCLC patients, with the data of 4.1 months for smokers and 1.9 months for never smokers,1.8 months for V600E and 4.1 months for other *BRAF* mutations; however there were only around 5% patients in the first line setting (3). The *BRAF* V600E non-smoking NSCLC patients left unsolved the question of the place of immunotherapy, especially in first line setting. In this study, we report a TKI naïve non-smoker female with a lung adenocarcinoma driven by *BRAF* V600E mutation concomitant with high PD-L1 expression treated with atezolizumab in combination with chemotherapy followed by BRAFi Vemurafenib, which could open a new perspective of treatment for non-smoking *BRAF* mutated lung cancer.

## Materials And Methods

The tissue biopsy at diagnosis was detected by BRAF V600E/EGFR/ALK/ROS1 Mutations Detection Kit (AmoyDx^®^). PD-L1 expression was confirmed by immunohistochemical (IHC) assay using E1L3N (Cell Signaling Technology, Danvers, MA). The cell-free DNA (cfDNA) at diagnosis and the tumor DNA and cfDNA at progression on immunotherapy and BRAFi were analyzed by next-generation sequencing (NGS) including 457 genes (BerryOncology Inc.). Objective tumor response was measured by computed tomography (CT) using RECIST v1.1criteria. The patient provided written informed consent authorizing publication of clinical case.

## Case Presentation

**Figure 1:**
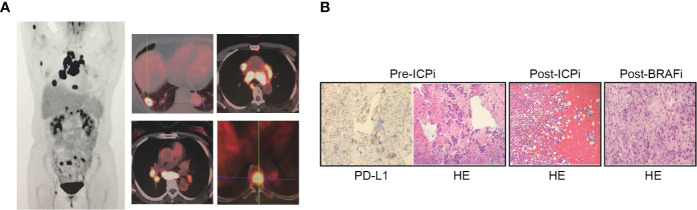
PET/CT imaging and histological finding. **(A)** PET/CT imaging on 2018/03/19 revealed an enlarged nodule measuring 2.0 × 1.6 cm on right lower lobe (RLL) with SUVmax 8.4, bilateral supraclavicular lymph nodes (0.5 cm and 0.6 cm on the right and left sides respectively) with SUVmax 2.8, enlarged mediastinal lymphadenectases including station 2R, 4R, 4L, 3A, 3P, 5, 6, 7, and right hilar lymphadenectasis with SUVmax 26.4 of the largest diameter of 4.2 cm, multiple bony metastases including right humerus, right scapula, T5 and T8 vertebral body, multiple sacrum, right acetabulum, right sciatic bone with a SUVmax 7.8. **(B)** Histological examination on the metastatic subcarinal lymphadenectasis tissue of pre-ICPi (first-biopsy), pericardial effusion of post-ICPi/pre-BRAFi (second-biopsy), and left supraclavicular lymphadenectasis of post-BRAFi (third biopsy) (hematoxylin and eosin stain, magnification ×100) and programmed death ligand-1 (PD-L1) expression in pre-ICPi (immunohistochemical stain, magnification ×100; E1L3N, Cell Signaling Technology, Danvers, MA); poorly differentiated lung adenocarcinoma positive for CK, TTF1, and NapsinA, negative for CD56 and P40 by immunohistochemistry stain in pre-ICPi (03/28/2018), post-ICPi/pre-BRAFi (12/20/2019), and post-BRAFi (06/04/2020). ICPi, immune check-point inhibitor; BRAFi, BRAF inhibitor.

A 62-year-old female non-smoker experienced a month-lasting fatigue, and PET/CT demonstrated an enlarged lung nodule on right lower lobe, bilateral supraclavicular and mediastinal lymphadenectases and multiple bony metastases confirming stage IV-T1bN3M1c (**Figure 1A**). Endobronchial ultrasound-guided transbronchial biopsy on subcarinal lymph node on 2018/03/28 revealed poorly differentiated lung adenocarcinoma with co-expressing tumors of *BRAF* V600E and PD-L1 ≥50% (**Figure 1B**). The concomitant liquid biopsy using NGS confirmed *BRAF* V600E mutation (**Supplementary Table 1**).

**Figure 2:**
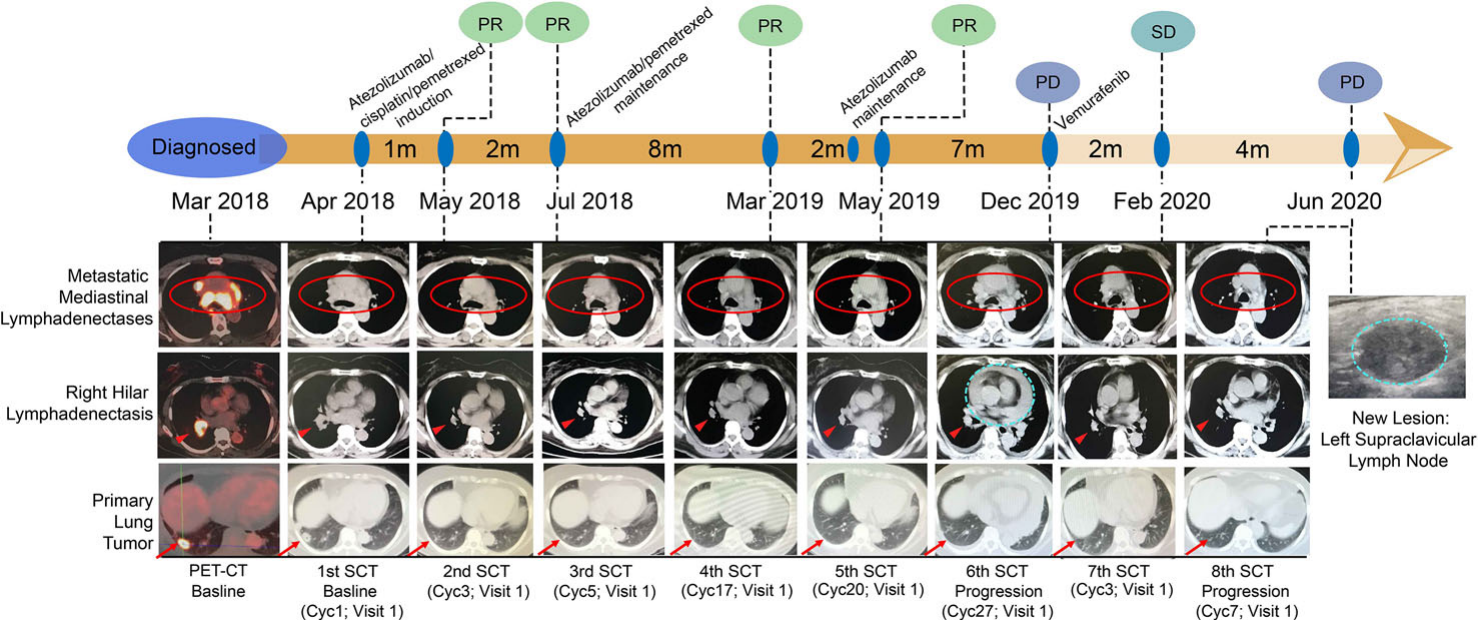
Course of the disease, treatment history, and response evaluation. 1st surveillance CT (SCT): baseline, atezolizumab/cisplatin/pemetrexed induction (Cyc1, 2018/04/11); 2nd SCT: post-induction two cycles (Cyc3, 2018/05/28), PR; 3rd SCT: post-induction four cycles, atezolizumab/pemetrexed maintenance (Cyc5, 2018/07/12), PR; 4th SCT: post-atezolizumab/pemetrexed maintenance 12 cycles (Cyc17, 2019/03/25), PR; 5th SCT: post-atezolizumab maintenance one cycle (Cyc20, 2019/05/27), PR. NOTE: Atezolizumab maintenance on C19V1. Tumor assessment every 9 weeks (three cycles) after the completion of the week 48 tumor assessment per protocol (Cyc20, 2019/05/27); 6th SCT: post-atezolizumab maintenance eight cycles (Cyc27, 2019/12/17), PD; 7th SCT: post-Vemurafenib two cycles (Cyc3, 2020/02/03), SD; 8th SCT: post-Vemurafenib six cycles (Cyc7, 2020/06/04), PD. Red circles: metastatic mediastinal lymphadenectases; Red short arrows: right hilar lymphadenectasis; Red long arrows: primary lung tumor; Blue dotted cycle: new lesion; lesion 1: pericardial effusion (progression on ICPi); lesion 2: left supraclavicular lymph node (progression on BRAFi). PR, partial response; SD, stable disease; PD, progressive disease; ICPi, immune check-point inhibitor; BRAFi, BRAF inhibitor.

**Figure 3:**
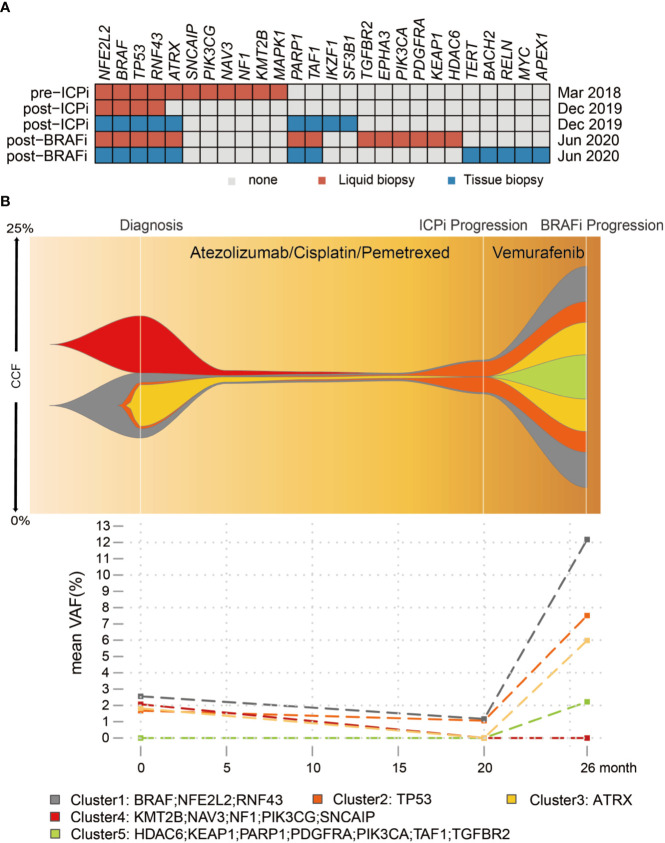
Somatic mutation profiles of pre-ICPi, post-ICPi/pre-BRAFi, and post-BRAFi. **(A)** The heatmap shows an overview of mutations in pre-ICPi, post-ICPi/pre-BRAFi, and post-BRAFi within liquid biopsy, pericardial effusion biopsy, and left supraclavicular lymph node. **(B)** Clonal evolution in liquid biopsy dynamic monitoring (Upper) and mean variant allele frequency (VAF) change (Lower) during immunotherapy and BRAF inhibitor Vemurafenib treatment. Variants from cfDNA using Bayesian cluster with PyClone. CCF, cancer cell fraction; ICPi, immune check-point inhibitor; BRAFi, BRAF inhibitor.

The patient enrolled in the trial IMpower132 (NCT02657434) and randomly assigned into the combination group was given atezolizumab/cisplatin/pemetrexed every three weeks during four-induction cycle period before atezolizumab/pemetrexed maintenance. The first cycle of atezolizumab (1,200 mg)/cisplatin/pemetrexed in the induction period was commenced on 2018/04/18. The patient experienced pyrexia (40°C) and grade III gastrointestinal toxicity, leading to a 25% dose reduction of chemotherapy from the second cycle, but without dose interruption or reduction of atezolizumab per protocol. A partial response (PR) of 57.3% including 60% of primary lung and 56.0% of metastatic lymph nodes was observed on first CT-scan evaluation after two cycles of atezolizumab in combination with cisplatin/pemetrexed treatment (**Figure 2**), and a continued PR was achieved for the following 14 cycles of atezolizumab/pemetrexed maintenance. Patient received atezolizumab maintenance from the 19^th^ cycle on 2019/05/09 considering grade II AST and ALT elevation. Unfortunately, disease progressed with the new pericardial effusion on 2019/12/17 after eighth cycles of atezolizumab maintenance, with a total of 20-months PFS of first-line treatment (**Figure 2**). The patient was treated with second-line treatment of BRAFi Vemurafenib (960 mg bid po) on 2019/12/20 and disease showed stable disease (SD) after two cycles. Unfortunately, the disease continued to progress with the new left supraclavicular lymphadenectasis but still with the controllable primary lung after 5.5 months. NGS showed the blood-based tumor mutation burden (bTMB) was decreased from 9.63/Mb at diagnosis to 3.50/Mb at post-ICPi-progression and bounced off to 10.51/Mb at post-BRAFi-progression (**Figure 3A** and **Supplementary Table 1**). The variant allele fraction (VAF) of mutation clusters in liquid biopsy was dropped at post-ICPi-progression compared with pre-ICPi; conversely, it drastically increased (excluding cluster 4) and new mutations occurred at post-BRAFi-progression compared with pre-/post-ICPi (**Figure 3B**).

## Discussion

Nowadays the therapeutic strategies of *BRAF*-mutated NSCLC are often introduced from melanoma treatment regimes. Due to the insufficiency of the study on *BRAF*-mutated subgroup in immunotherapy trials in NSCLC, which therapeutic strategy, immunotherapy, or targeted therapy was used as first-line remains an issue. Patients with *BRAF* V600E melanoma had longer PFS than those with *BRAF* V600K melanoma in targeted-therapy, and conversely the shorter PFS in the immunotherapy, providing the differences in gene expression and mutational load between V600E and V600K *BRAF*-mutant melanomas with the differences in response to BRAFi−/+ MEKi inhibitors and immunotherapy (4). Immuno-target therapy might work better for *BRAF*-mutant NSCLC smokers and seldom beneficial to non-smokers and light smokers (3). Indeed, never-smoking status with a targetable driver is usually accompanied with low TMB and low PD-L1 expression, which might partially explain the low efficacy of ICPi (5). While a retrospective study reported that all eight Asian lung cancer patients with *BRAF* V600E and PD-L1 ≥50% had co-expressing tumors, six were non-smokers (6), which is unlikely the same as that in Caucasian population. Co-expressing tumors account for 42% in NSCLC west Asian population (6) that is much higher compared to 28% in NSCLC with around 70% Caucasian population (7), which may partially explain the difference of immunotherapy efficacy in *BRAF* non-smoking NSCLCL. In ICPi/chemotherapy NSCLC clinical cohorts (8–13), combination groups demonstrated longer median PFS or OS than chemotherapy alone groups. Especially, the trials KEYNOTE-189, IMpower150, and IMpower130 which investigated the patients with previously untreated metastatic non-squamous NSCLC also displayed that the combination treatment was superior to single-chemotherapy. Furthermore, the magnitude of PFS and OS benefit with ICPi/chemotherapy combination correlated well with PD-L1 and was observed in these cohorts (8–13). The similarities of these cohorts and our case are that patients were EGFR/ALK negative, and some of them presented high PD-L1 expression. Here we reported the first-line combination of atezolizumab with chemotherapy improved PFS to 20 months in a non-smoking *BRAF* V600E-mutated NSCLS patient. The comparison of the result of the IMMUNOTARGET registry study showed median PFS was 1.8 months with ICPi treatment for V600E in the later line which is much shorter than that in the first line.

Interestingly, in our case, bTMB dropped at progression on immunotherapy, implying immunotherapy resistance occurred as the result of the loss of neoantigen (14). At progression, *BRAF* V600E mutation was both identified in pericardial effusion (new lesion) and plasma. A *PARP1* mutation occurred in pericardial effusion lesion at progression on the combination of immunotherapy and chemotherapy, though no direct evidence has pointed out the relationship between *PARP1* mutation and immunotherapy resistance yet. However, new finding shows that PARP inhibition can synergy with ICPi (15). PARP inhibition regulates the DNA damage response pathway and accumulates DNA injuries, which may lead to the accumulation of neoantigens for ICPi (16). In addition, the research of PARP-inhibitor-mediated upregulation of PD-L1 has been reported (17).

There was a median PFS of 5.2 months in 100 *BRAF* V600-mutated NCSLC patients treated with Vemurafenib found by J. Mazieres et al. (1) and our case with around 5.5 months. BRAFi therapy yields high ORR, while therapeutic duration is limited by diverse mechanisms of acquired resistance. Our result showed a rapid increase of VAF of all mutation clusters (excluding cluster 4) during BRAFi treatment to progression, and reactivation of the MAPK/Erk pathway is a major contributor to BRAFi treatment failure. A *PDGFRα* G853D mutation was found after BRAFi treatment in liquid biopsy; PDGFR is upstream of RAS in the MAPK pathway and strong autophosphorylation was observed in PDGFR*α* G853D mutation (18). Deregulation of the PI3K pathway which was found out by the presence of *PI3KCA*, *AKT1*, *PTEN*, or *PPP2R1A* mutations can cause resistance to BRAFi. Our case showed *PI3KCA* mutations after BRAFi progression. Furthermore, the genomic alterations in *PIK3CA* have been depicted in resistance to BRAFi in *BRAF* V600E melanoma and NSCLC (19).

## Conclusion

This case shows the durable response to the combination of atezolizumab with chemotherapy and the prolonged PFS of 20 months in an untreated patient with non-smoking *BRAF* V600E-mutated lung adenocarcinoma, supporting the idea that combination immunotherapy may be an attractive option for *BRAF* V600E non-smoking NSCLC with high PD-L1 expression patients. This case highlights the need for further research to explore the efficacy of immuno-target therapy and ICPi in correlation with different molecular parameters in *BRAF* mutant NSCLC.

## Data Availability Statement

The datasets presented in this study can be found in online repositories. The names of the repository/repositories and accession number(s) can be found in the article/**Supplementary Material**.

## Ethics Statement

The studies involving human participants were reviewed and approved by Shanghai Chest Hospital. The patients/participants provided their written informed consent to participate in this study. Written informed consent was obtained from the individual for the publication of any potentially identifiable images or data included in this article.

## Author Contributions

Design and supervision of this study: XN, SL, and XA. Data collection and analysis: XN, YS, LC, and JB. Manuscript writing: XN, DP, and LC. Final approval: XN, YS, DP, LC, JB, XA, and SL. All authors contributed to the article and approved the submitted version.

## Funding

This work was supported by grants from the National Natural Science Foundation of China (No. 81972187), Projects of the Committee of Shanghai Science and Technology (Nos. 19ZR1449800, 20Y11913700, 19411950500, 18DZ1910702), National Key Research and Development Program of China (No. 2016YFC1303300), Clinical Research Project of Shanghai Municipal Public Health Bureau (No. 201840122), Advanced Appropriate Technology Promotion Project of Shanghai Municipal Public Health Bureau (No. 2019SY048), Doctoral Innovation Fund of Shanghai Jiao Tong University School of Medicine (No. BXJ201952), and Interdisciplinary Program of Shanghai Jiao Tong University (No. YG2017MS80),Project of Shanghai Talent Development Fund (No. 2019074).

## Conflict of Interest

LC and JB were employed by the company Berry Oncology Corporation.

The remaining authors declare that the research was conducted in the absence of any commercial or financial relationships that could be construed as a potential conflict of interest.
