# Supplementary material for: Durable Response to the Combination of Atezolizumab With Platinum-Based Chemotherapy in an Untreated Non-Smoking Lung Adenocarcinoma Patient With BRAF V600E Mutation: A Case Report
Source: Front Oncol. 2021 Jun 10;11:634920. doi: 10.3389/fonc.2021.634920 (PMC8222507; doi:10.3389/fonc.2021.634920)
Supplement: Supplementary file 1 [file Table_1.docx]

[**Supplementary Table 1**](https://oup.silverchair-cdn.com/oup/backfile/Content_public/Journal/annonc/30/6/10.1093_annonc_mdz055/1/mdz055_supplementary_data.zip?Expires=1578639729&Signature=QhVCJZ~U20qZTUBC-ZIyiGIspjl8V9D~A3zH3NSNsqEnWAugwbnX3FS~ivoVKYdVZ-SaDzWLQdk9cDzpPnNgawLyMhImT0zs56CWtaLicb3gLTv2RaarhzGV~X5uaPDQJ10RWcKfCnQ1dcBMcagvHjGlVJci6ro9gkjmUUayECuHV~HXzn754tv1btjD~BVgHgTWxN81SyfRg7kSCe1DBRSK4zlC-TjRba2gIj9T9B1dBHIz1VKEdscul-TlK3wSDeI2h4VDUwCnmDE6WO4cJMaPnxx~6qIi2KMXraSn28-2jmTFk~Y-PfCU1oA08RjY~39ZtcYGX31bHKb9TSQl4Q__&Key-Pair-Id=APKAIE5G5CRDK6RD3PGA)**.** Mutations identified in liquid biopsy, pericardial effusion, and left supraclavicular lymph node using next-generation sequencing (NGS) analysis in pre-ICPi, post-ICPi/pre-BRAFi, and post-BRAFi treatment.

|  | **Gene** | **DNA change** | **Amino acid change** | | **VAF**  **(%)** | **Type** |
| --- | --- | --- | --- | --- | --- | --- |
| **Mar 2018**  **(pre-ICPi)** | ***cfDNA*** | | | | | |
|  | NFE2L2 | c.92G>C | p.G31A | | 2.58 | nonsynonymous SNV |
|  | SNCAIP | c.787T>C | p.F263L | | 1.56 | nonsynonymous SNV |
|  | PIK3CG | c.1316C>T | p.P439L | | 3.93 | nonsynonymous SNV |
|  | BRAF | c.1799T>A | p.V600E | | 3.06 | nonsynonymous SNV |
|  | NAV3 | c.600G>C | p.Q200H | | 1.77 | nonsynonymous SNV |
|  | TP53 | c.1040C>T | p.A347V | | 1.67 | nonsynonymous SNV |
|  | NF1 | c.4700C>G | p.S1567X | | 0.55 | stopgain |
|  | RNF43 | c.935G>A | p.C312Y | | 2.03 | nonsynonymous SNV |
|  | KMT2B | c.7177G>A | p.E2393K | | 2.53 | nonsynonymous SNV |
|  | ATRX | c.6160C>A | p.L2054I | | 1.82 | nonsynonymous SNV |
|  | MAPK1 | Copy number: 3.10 | | | | Amplification |
|  | bTMB:9.63/Mb | | | | | |
| **Dec 2019**  **(post-ICPi/pre-BRAFi)** | ***cfDNA*** | | | | | |
|  | BRAF | c.1799T>A | p.V600E | | 0.92 | nonsynonymous SNV |
|  | TP53 | c.1040C>T | p.A347V | | 1.06 | nonsynonymous SNV |
|  | NFE2L2 | c.92G>C | p.G31A | | 1.42 | nonsynonymous SNV |
|  | RNF43 | c.935G>A | p.C312Y | | 1.16 | nonsynonymous SNV |
|  | bTMB: 3.50/Mb | | | | | |
|  | ***pericardial effusion*** | | | | | |
|  | ATRX | c.6160C>A | p.L2054I | | 18.14 | nonsynonymous SNV |
|  | BRAF | c.1799T>A | p.V600E | | 35.72 | nonsynonymous SNV |
|  | IKZF1 | c.727G>A | p.D243N | | 8.38 | nonsynonymous SNV |
|  | NFE2L2 | c.92G>C | p.G31A | | 51.37 | nonsynonymous SNV |
|  | PARP1 | c.853G>A | p.D285N | | 11.07 | nonsynonymous SNV |
|  | RNF43 | c.935G>A | p.C312Y | | 42.09 | nonsynonymous SNV |
|  | SF3B1 | c.762G>A | p.W254* | | 16.49 | stopgain |
|  | TAF1 | c.2680G>A | p.E894K | | 24.54 | nonsynonymous SNV |
|  | TP53 | c.1040C>T | p.A347V | | 60.15 | nonsynonymous SNV |
|  | TMB: 7.88/Mb | | | | | |
| **June 2020**  **(post-BRAFi)** | ***cfDNA*** | | | | | |
|  | ATRX | c.6160C>A | | p.L2054I | 5.98 | nonsynonymous SNV |
|  | BRAF | c.1799T>A | | p.V600E | 12.53 | nonsynonymous SNV |
|  | EPHA3 | c.1346C>T | | p.S449F | 1.85 | nonsynonymous SNV |
|  | HDAC6 | c.830G>A | | p.R277H | 1.33 | nonsynonymous SNV |
|  | KEAP1 | c.1136G>A | | p.G379D | 0.19 | nonsynonymous SNV |
|  | NFE2L2 | c.92G>C | | p.G31A | 12.98 | nonsynonymous SNV |
|  | PARP1 | c.853G>A | | p.D285N | 4.70 | nonsynonymous SNV |
|  | PDGFRA | c.2558G>A | | p.G853D | 0.17 | nonsynonymous SNV |
|  | PIK3CA | c.1357G>C | | p.E453Q | 0.18 | nonsynonymous SNV |
|  | RNF43 | c.935G>A | | p.C312Y | 11.02 | nonsynonymous SNV |
|  | TAF1 | c.2680G>A | | p.E894K | 8.14 | nonsynonymous SNV |
|  | TGFBR2 | c.1051G>A | | p.A351T | 0.82 | nonsynonymous SNV |
|  | TP53 | c.1040C>T | | p.A347V | 7.51 | nonsynonymous SNV |
|  | bTMB:10.51/Mb | | | | | |
| **June 2020**  **(post-BRAFi)** | ***left supraclavicular lymph node*** | | | | | |
|  | APEX1 | c.356G>C | | p.W119S | 21.50 | nonsynonymous SNV |
|  | ATRX | c.6160C>A | | p.L2054I | 15.97 | nonsynonymous SNV |
|  | BACH2 | c.2352C>A | | p.S784R | 45.48 | nonsynonymous SNV |
|  | BRAF | c.1799T>A | | p.V600E | 31.86 | nonsynonymous SNV |
|  | MYC | c.1273G>A | | p.E425K | 14.61 | nonsynonymous SNV |
|  | NFE2L2 | c.92G>C | | p.G31A | 55.23 | nonsynonymous SNV |
|  | PARP1 | c.853G>A | | p.D285N | 15.43 | nonsynonymous SNV |
|  | RELN | c.9187G>A | | p.D3063N | 13.74 | nonsynonymous SNV |
|  | RNF43 | c.935G>A | | p.C312Y | 40.74 | nonsynonymous SNV |
|  | TAF1 | c.2680G>A | | p.E894K | 18.23 | nonsynonymous SNV |
|  | TERT | c.902G>A | | p.R301H | 10.18 | nonsynonymous SNV |
|  | TP53 | c.1040C>T | | p.A347V | 51.81 | nonsynonymous SNV |
|  | TMB: 8.78/Mb | | | | | |

**Note:**

(1) Panel information: The NGS panel was designed to detect single nucleotide variations (SNVs), small insertions and deletions (indels), gene rearrangements, and copy number variants (CNVs) by covering entire coding sequence (CDS) regions of 456 unique genes and selected introns.

(2) Method: Mapping and quality control were performed by bwa mem ^[1]^ and flexbar ^[2]^, respectively. Reads with same barcodes, same start/stop were marked as PCR duplications and biases and sequence errors which were eliminated to generate consensus reads. Somatic SNVs and indels were identified by in-house developed mutation calling tool, MutLoc. The calculation of region-based fragments was employed by GATK3.6, gene-based CNVs were attributed to region-based gains and loss. The candidate gene fusions were detected from junction spanning reads, candidates with ≥2 junction reads were retained and filtered depending on junction sequences. TMB is calculated as below: TMB= (total number of nonsynonymous and synonymous mutations– the number of hotspot mutations)/ panel size in Mb.

(3) Abbreviation: ICPi: immune check-point inhibitor; BRAFi: BRAF inhibitor.

**Reference:**

[1] Li H, Durbin R. Fast and accurate short read alignment with Burrows-Wheeler transform. *Bioinformatics*. 2009;25(14):1754-1760.

[2] [Roehr JT](https://www.ncbi.nlm.nih.gov/pubmed/?term=Roehr%20JT%5BAuthor%5D&cauthor=true&cauthor_uid=28541403), [Dieterich C](https://www.ncbi.nlm.nih.gov/pubmed/?term=Dieterich%20C%5BAuthor%5D&cauthor=true&cauthor_uid=28541403), [Reinert K](https://www.ncbi.nlm.nih.gov/pubmed/?term=Reinert%20K%5BAuthor%5D&cauthor=true&cauthor_uid=28541403). Flexbar 3.0 - SIMD and multicore parallelization. *Bioinformatics*. 2017;33(18):2941-2942.
